# Does small and medium enterprise differential leadership increase subordinate knowledge hiding? Evidences from job insecurity, territorial consciousness and leadership performance expectation

**DOI:** 10.3389/fpsyg.2022.983669

**Published:** 2022-09-16

**Authors:** Jing Xu, Dequn Zhu, Yongzhou Li

**Affiliations:** ^1^Evergrande School of Management, Wuhan University of Science and Technology, Wuhan, China; ^2^School of Economics and Management, Shangrao Normal University, Shangrao, China

**Keywords:** SME differential leadership, subordinate knowledge hiding, job insecurity, territorial consciousness, leadership performance expectation

## Abstract

Leadership is considered as a significant antecedent of knowledge hiding in SMEs (small and medium enterprises), but the differential dimension of leadership has been evidently neglected in both theoretical and empirical areas. Drawing on conservation of resource theory and social cognitive theory, this research investigates whether and how SME differential leadership influences subordinate knowledge hiding. Specifically, we analyze the underlying mechanisms of a chain-mediator—job insecurity and territorial consciousness and a boundary condition—leadership performance expectation. Multi-wave and multi-source data were collected from a sample of 704 Chinese SME employees and 140 relevant leaders and applied HLM meso-mediational frameworks, and Bootstrap technique with non-parametric percentile residuals for deviation correction. The results show that differential leadership plays a potential role in promoting subordinate knowledge hiding through the serial intervening mechanism of job Insecurity and territorial consciousness in SMEs. Furthermore, the positive relationship between SME differential leadership and job insecurity becomes stronger among subordinates under higher leadership performance expectation; the positive indirect relationship between SME differential leadership and subordinate knowledge hiding is stronger with higher levels of leadership performance expectation. This study contributes to the existing academic literature by empirically analyzing the under-investigated correlation between differential leadership and subordinate knowledge hiding in SMEs, and by exploring the underlying mechanisms and a boundary condition.

## Introduction

In the post-COVID-19 global industry landscape, leadership inevitably guides the survival and development of small and medium enterprises (SMEs). SMEs are complex and dynamic operational systems (Cortes and Herrmann, 2020; Hussain et al., 2020; Huang et al., 2022). To form to long-term competitive and developmental advantages, SMEs need to rely on the “trickle-down effect” from excellent leadership. The trickle-down effect is one of the new paradigms of leadership management (Corwin et al., 2022), which is “on the spilling effect from top to bottom” (Ullah et al., 2022, p. 46). A wealth of academic research has suggested that leadership is a vital predictor of prosocial behaviors (Lee and Chon, 2020; Mustofa and Muafi, 2021), because superior-subordinate interactions not only influence individual cognition on job role positioning, but also affect individuals’ proactive behaviors by shaping organizational structures and cultural values (Mo and Shi, 2017; Bavik et al., 2018). Prior research has examined the impacts of various leadership traits and strategies on constructive behaviors, extra-role behaviors or pro-social behaviors, including traditional ones such as authoritarian leadership (Jiang et al., 2017; Guo et al., 2018), transformational leadership (Den Hartog and Belschak, 2012; Breevaart et al., 2016), servant leadership (Panaccio et al., 2015), inclusive leadership (Randel et al., 2018; Cenkci et al., 2020), and emerging ones such as paradoxical leadership (Li et al., 2018; She et al., 2020), authentic leadership (Agote et al., 2015), ethical leadership (Wen et al., 2021), self-sacrificing leadership (Hoogervorst et al., 2012), mindfulness leadership (Khalil et al., 2021), and superiors’ spiritual leadership (Afsar et al., 2016), to name a few. These aforementioned leadership studies have made obvious contributions to our understandings of how organizational leadership affects pro-social initiatives. However, the differential classification of leadership has been neglected in the context of pro-social knowledge sharing.

Rather than only focusing on the traits or actions of leadership in certain appointed positions, differential leaders pay special attention to followers’ loyalty and talent to discriminate them as “insiders” or “outsiders” accordingly, and how leaders create relationships with diverse groups (Tang et al., 2018; Wang et al., 2018). Differential leadership “favors insiders over outsiders in their management and resource allocation (Wu et al., 2021, p. e9746).” This definition indicates that differential leadership consists of relational values including a sender-receiver concept that incorporates differentiated arrangements of work resources as well as accepting responses from insiders and outsiders (Li and Xie, 2022). This is highly relevant to the concealment of knowledge, as knowledge hiding is “anti-pro-social activities of withholding knowledge” whereby fictitious ignorance, evasive concealment or rational hiding toward personal intellectual capital to prevent ego interests and ownership from being occupied by others (Serenko and Bontis, 2016; El-Kassar et al., 2022; Rubbab et al., 2022). The association between differential leadership and knowledge hiding validates the importance of leadership oriented to favoring insiders in resource allocation and support. That is, the insiders tend to deliberately perform counterproductive knowledge behaviors where others consults them for valuable intellectual capital, while the outsiders do not own enough intellectual assets, facing the failure of sharing their knowledge (Donate et al., 2022; Jafari-Sadeghi et al., 2022). Notwithstanding the potential impediment that differential leadership brings to knowledge exchange and flow, little theoretical and empirical study has been conducted on whether and how differential leadership affects subordinate knowledge hiding.

To address these issues, this research, based on conservation of resource theory and social cognitive theory, explores whether and how differential leadership affects subordinate knowledge hiding within SMEs. According to conservation of resource theory, SME employees have scarce (for outsiders) or heterogeneous (for insiders) intellectual resources (French et al., 2015), and especially, study has demonstrated that differentiated leadership can impede personal knowledge sharing (Gelens et al., 2014; Sembiring et al., 2020). By damaging individual motivations to engage in information interaction and communication, differential superiors may weaken interpersonal relationships with trust, develop increased conflict, and slow down information exchange and resource sharing in terms of knowledge-intensive industry (Yeşil and Dereli, 2013; Tan et al., 2022), which accelerate the knowledge hiding process. In addition, social cognitive theory has opened up a new path for social learning process (Scott et al., 2022). Employees bear some negative impacts (e.g., knowledge hiding behavior) from the antecedent of differential leadership management through a series of psychological dynamics caused by a person’s information-processing system, which consists of five dimensions, including coding, explanation, search response, reaction evaluation, and reaction practice (Rakib et al., 2022). Hence, exploring a new insight into the link between differentiated management and knowledge hiding is needed.

To elaborate on how the influence process (SME differential leadership → subordinate knowledge hiding) unfolds, we introduce job insecurity and territorial consciousness as a chain mediating mechanism. Conservation of resource research has been expected to explore underlying processes through which resource allocation characteristics affect receivers’ responses (Chen et al., 2015). The latest reviews on conservation of resource theory state that perceived stress or psychological ownership can act as the crucial underlying mechanisms to unpack the “black box” between resource allocation characteristics and distal receivers’ behaviors (Chang et al., 2012; Singh et al., 2019). Job insecurity—the extent to which organizational employees perceive stress to maintain the continuity of the status quo and worry about threatened powerlessness (Vander Elst et al., 2014; Vieira Dos Santos et al., 2022)—may be stimulated by unconventional superior-subordinate interactions. Territorial consciousness represents the reinforcement of psychological ownership, which is largely triggered by senses of possession and affects employee behavioral strategies for specific targets (Jussila et al., 2015; Chen et al., 2020). Furthermore, territorial awareness has been acknowledged to bring great impacts on knowledge sharing because it enhances interactive resistance, emphasizes self-ownership, and brings about defensive mentality (Avey et al., 2012; Peng and Pierce, 2015). Given the upper positions of SME leaders, their differential treatment toward subordinates can have a significant effect on the employees’ insecurity and territorialism related to knowledge hiding decisions.

In addition, this study explores a boundary condition to extend the understandings of how SME differential leadership lead to subordinate job insecurity and knowledge hiding. How employees interpret and respond to external leadership characteristics depends on their cognition states shaped by superior instructions, such as leadership performance expectation (Li et al., 2019). When differential upper management signals that they are concerned or careless about the quality and excellence of subordinates’ performance, subordinates may feel different levels of stress. Prior research has demonstrated that enterprise employees with higher excellence performance expectation need to hold and invest more resources to pursue sustainable performance (Chen and Liang, 2017; Jacobsen and Andersen, 2017; Wang et al., 2020). Thus, SME employees with higher performance anticipation are more likely to be motivated by differential leaders to advance individual perception of occupation insecurity, which may then promote subordinate knowledge hiding.

We broaden the existing literature in followings perspectives. First, surprisingly, the potential influence of SME differential leadership on knowledge-related activities has received relatively little attention. It is of greater significance to fill such an unexplored gap, as knowledge has played as a fundamental prerequisite for SME survival and development in the knowledge economy era (Jafari-Sadeghi et al., 2022). Consistent with extensive evidence that differential leadership triggers a host of dysfunctional actions (French et al., 2015), and emerging literature calls for more attention for triggers of deliberate concealment on intellectual self-capital (Rakib et al., 2022), we infer that differential leadership may also evoke knowledge hiding, and propose the study issue that does SME differential leadership increases subordinates’ knowledge hiding? Specifically, based on the framework constructed by conservation of resource theory and social cognitive theory, how do five dimensions of psychological dynamics caused by SME differential leadership individually influence knowledge hiding? Second, this study examines how to transmit the negative impact of SME differential leadership by introducing job insecurity and territorial consciousness as a chain-mediating variable. When SME employees perceive high occupational insecurity, this insecurity threaten subordinates’ diminished control over own selves, jobs, and resources, increasing territorial consciousness. Under the mental state of territorial consciousness, these SME employees would choose to hide knowledge to reflect their discomfort. Third, by identifying a key conditional factor—leadership performance expectation, this research also expands the understanding of how SME leadership facilitates job insecurity or knowledge hiding. Although prior study has noted the negative effects of differential leadership on individual outcomes, it has ignored the explorations on boundary conditions. Under higher leadership performance expectation, SME employees have to invest more time, energy, individual tangible and intangible resources into more challenging tasks. Leadership performance expectation virtually strengthens the emotional and belonging dependence on knowledge resources and can deepen the uneasiness at work caused by the demand of defending successive losses of self-resources. Then the differential leadership is more likely to cause job insecurity and knowledge hiding. Our research propositions provide practical reference for small and medium enterprises to reduce knowledge hiding activities.

## Theoretical background and hypotheses

### Theoretical background

As the upper layer of the pyramid structure in SMEs, leadership has an outsize impact on employees’ behavior motivations; this has been widely confirmed from various angles, including leadership styles, management strategies, internal control, etc. (Saridakis et al., 2013; Cortes and Herrmann, 2020; Tolulope et al., 2020). In particular, prior study has explored various types of SME leadership and the influences on employee extra-role or constructive behaviors. For example, scholars have indicated that SME servant leadership benefits proactive socialization outcomes by triggering employees’ job satisfaction, psychological contract and person-job fit (Panaccio et al., 2015; Bauer et al., 2019). Rasheed et al. (2017) specified encouraging leadership—a leadership style in high-performance work systems, and showed that SME encouraging superiors can motivate employee voice and task participation by setting and conveying organizational visions. Banwo and Du (2019) pointed out that perceived control from leadership can promote employee pro-environmental behaviors by establishing social norms within SMEs. Abdullahi et al. (2020) confirmed that SME employees’ organizational citizenship behavior (OCB) is inspired by democratic and transformational leadership, which both refer to supportive leadership styles and entail the followings: authorization and creativity, respectively.

Although the existing study has made obvious contributions in the area of SME leadership, most theories view leadership practices as a main development driver for SMEs and place various leader properties at the center of our understanding of SME leadership (Elshout et al., 2013). Particularly, the differential attribute of leadership deserves a more detailed interpretation (Jada and Mukhopadhyay, 2019; Wu et al., 2021; Zhang et al., 2022). According to Wang et al. (2018), differential leadership is “a leader who behaves differently toward his/her members by giving more support to in-groups over out-groups” (p. 1070). The difference-order leadership pattern means differential resource allocation processes in which Confucianism relationalism (e.g., in-group members with a clear boundary, favoritism treatment) and eastern traditional values (e.g., circle culture, culture of human feeling, familialism, communitarianism) are rooted. Furthermore, by defining the boundary of in-groups in the form of preference, unbalanced allocation, communication frequency, rewarding promotion, and fault-tolerance, differential leadership shows its unique characteristics (Knapp et al., 2014; Dai and Chen, 2015; Pervaiz et al., 2021; Metz et al., 2022). Although some traditional leadership concepts [e.g., leader-member exchange (LMX) theory] and differential leadership all emphasize role-making, trust-relationship and social-exchange, LMX theory, as one of the interpersonal studies, mainly captures the reciprocity core between superiors in leadership positions and their followers (Erdogan and Bauer, 2014), whereas according to personalized standards for benefiting the core circle, differential leadership places a greater focus on providing different resources and opportunities to in- versus out- groups.

Moreover, some scholars on individual knowledge management have called for more study on the impacts from the “leadership side” on knowledge owners’ interactive behaviors, that is, how knowledge owners construct relations with their superiors (Abdullah et al., 2019; Jasimuddin and Saci, 2022). Individual knowledge-based behaviors involve not only information sharing processes but also hiding ones (Ma et al., 2013). The differential leadership style undoubtedly affects employee knowledge-related exchanges in the era of intensifying competition environment. When organization employees receive distrust signals from their differential leaders, a self-preservation awareness that is critical for knowledge hiding may emerge (Malik et al., 2019). In addition, the resource superiority induced by leadership can also motivate subordinates to hide knowledge for its scarcity (Serenko and Bontis, 2016; Batistic and Poell, 2022). Despite these crucial antecedent mechanisms, the differential classification of leadership has been largely ignored in the context of knowledge hiding. The emphasis on the “leadership side” in knowledge hiding research is in line with the “relationality dynamics” in superior-subordinate interactions, which have attracted increasing attention (Khalid et al., 2018; Babic et al., 2019). Therefore, it is necessary to explore the relationship between differential leadership and subordinate knowledge hiding.

In this research, we adopt conservation of resource theory as the theoretical framework. Conservation of resource theory states that humans integrate external and cognitive elements to acquire, conserve, retain and plan resources, which, in turn, affects individuals’ behavioral tendencies, choices and decisions together with the associated psychological costs (Peck, 2021). Researchers have applied conservation of resource theory to analyze the influences of different types of SME leadership on personal behavior outcomes (Özer and Tınaztepe, 2014; Bojadziev et al., 2019; Cortes and Herrmann, 2020). To unpack the underlying mechanisms, scholars, furthermore, have focused on the mediating roles of individual psychological mechanisms, such as perception (Abdullahi et al., 2020) as well as consciousness (Banagou et al., 2021). In this paper, we select job insecurity and territorial consciousness as two aspects of individual mental characteristics to explain the underlying mechanisms of the “black box” between SME differential leadership and knowledge hiding. Meanwhile, leadership performance expectation is introduced into exploring its moderating effect.

### Small and medium enterprise differential leadership and job insecurity

Based on Feather and Rauter (2004), job insecurity can be defined as the extent to which enterprise members experience powerlessness, worry about accidents in current job, and lose desired continuity within an unpredictable working situation (Staufenbiel and König, 2010; Wang et al., 2015). Although few extant researches have examined the effect of SME differential leadership on job insecurity, an increasing number of analyses have found that differential leadership is inherently linked to obstructive stress from “favoritism” or “injustice” (Guerrero et al., 2013; Wang and Kim, 2013), which deviate from the fundamental needs of job stability and security.

Specifically, differential leadership emerges from the identification of insiders vs. outsiders (Dai and Chen, 2015; Caron et al., 2019). Resource distribution, which is endowed with independent standards characterized by the willingness to promote insiders’ career development, is one of the main forms of differential insider-outsider treatment (Horng et al., 2016). Being engaged in key resource allocation, SME differential leaders provide more scarce heterogeneous resources for insiders’ self-promotion (Caron et al., 2019). Such distribution increases in-group members’ vigilance of heterogeneous resources loss, which is positively related to insiders’ job Insecurity (Tang et al., 2018). Differential leaders in SMEs also convey unfavorable information to resource-limited outsiders to intensify worries about career prospects, which in turn increases their job insecurity (Smith et al., 2012).

Furthermore, SME differential superiors advance task assignment differences among insiders and outsiders; they take stock in in-group members extraordinarily and entrust them with core business (Knapp et al., 2014), which may bring great challenges and additive pressure on self-development, and engender insecurity in performing job tasks. This differentiated management mode also assign routine and laborious tasks to out-group individuals and make them feel dispensable and insignificant in the organization and foster a climate of task insecurity (Stamper and Masterson, 2002; Smith et al., 2012; Li et al., 2014). Based on the aforesaid arguments, this study proposes the following hypothesis:

*Hypothesis 1: SME differential leadership is positively related to job insecurity.*

### Job insecurity and territorial consciousness

Territorial consciousness is an indicator referring to individuals’ determination, insistence, and willpower in protecting what they own and value (Rechberg and Syed, 2013). A possession sense of territory involves two characteristics: an ownership sense of target objects (i.e., it is mine!), and an autonomous control toward ownership targets (Lee and Suh, 2015; Seo and Park, 2021). This research argues that job insecurity is crucial to the processes above. Insecurity from the own job emphasizes the job stress and anxiety among organizational members, which may guide them into the fear and worry of losing current resources. Job powerlessness and insecurity are stimulated by the challenges from averting loss events in the status quo (Probst et al., 2007). The sense of occupational insecurity advances potential conflicts between the two sides in employment relations and facilitates employees’ defense against job-related threats (Staufenbiel and König, 2010; Vander Elst et al., 2014; Stankevičiûtë et al., 2021).

Previous study has provided evidence that job insecurity is closely associated with cognitive preventative focus, safety self-regulation, obstructive stressors, and the strong sense of personal boundaries (Pittino et al., 2018; Wang et al., 2019; Park and Shin, 2020). Cognitive preventative focus and the setting of personal boundaries shows the resistance to exchanging tangible or intangible objects with the individuals outside the territory (Wang et al., 2019). Safety self-regulation and obstructive stressors can stimulate the territoriality idea sensitive to resource outflow (Hameed et al., 2019; Xia et al., 2019). Thus, subordinates with job insecurity are more likely to predict the appropriation of valuable objects and build territorial consciousness to retain and protect current self-resources firmly.

Therefore, People with higher insecurity perception have stronger psychological ownership for possessions and invest effort in ensuring job stability, controllability and predictability (Hameed et al., 2019); they are more likely to defend self territory to strengthen implicit self-identity based on factual ownership toward representational objects. Through a close connection with possessive targets, employees with job insecurity also view these targets as an extension of the self, and increase their motives to guarantee the continuity of belongingness (Yu et al., 2021). We put forth the following hypothesis:

*Hypothesis 2: A positive relationship exists between job insecurity and territorial consciousness.*

### Territorial consciousness and subordinate knowledge hiding

The implementation of knowledge hiding is a process of withholding private knowledge that involves the adoption of rationalized hiding or inevasive hiding to conceal accurate information (Connelly et al., 2012). On the one side, holders with poor knowledge who occupy unimportant positions in the organization suffer both a difficulty in gaining extrinsic knowledge and an ease of losing existing one (Černe et al., 2014). People with inadequate knowledge may concentrate more effort in knowledge management, which always leads them into an exhausted state. Hence, these individuals tend to decline knowledge requests from others intentionally (Banagou et al., 2021). In fact, it is also difficult for knowledge-lack employees to abandon ownership perception and engage them in knowledge sharing. Employees owning little knowledge often feel unconfident and accept the territorial awareness, thoughts, and beliefs regarding resource conservation to realize self-worth and knowledge concealment-enhancing (Connelly and Zweig, 2015; Demirkasimoğlu, 2016). Subordinates holding a small amount of knowledge are more likely to construct a self-enclosed psychological state where knowledge flows and information exchange are avoided, and have an urgent need to hide knowledge resources.

On the other hand, employees approaching heterogeneous knowledge may have a territorial concept that emphasizes self-centered and self-derived defense and lowers sharing of valuable knowledge (Anand and Jain, 2014; Serenko and Bontis, 2016). Employees with high knowledge reserves, who usually pay much attention to the attainment of personal goals, career growth or optimal development (Černe et al., 2014), believe that they can create better job performance by planning and applying the existing heterogeneous knowledge (Ladan et al., 2017). After a long-term and hard accumulation, employees who have enough knowledge reserves have completed higher-level self-realization in intellectual capital, so their knowledge management patterns encompass maintenance and active defense principles, that is, with high knowledge stocks, knowledge management patterns tend to be loss-prevention (Cegarra-Navarro et al., 2021). A knowledgeable organizational individual prefers to adopt sharing avoidance strategies for knowledge, and believe that guarding personal resource territory is more important than expanding it blindly (Anand and Jain, 2014; Liu et al., 2020).

In general, personal territorial consciousness promotes the generation of resource-hiding tendencies, which triggers an unwillingness to share professional knowledge, opinions, ideas or insights (Avey et al., 2012; Connelly et al., 2012; Peng and Pierce, 2015). Literature has argued that knowledge hiding intentions are often embedded in territorial consciousness (Wang et al., 2019) because territorial autonomy underlines a continuous possession consciousness to maintain and protect intangible knowledge objects with determination (Demirkasimoğlu, 2016; Banagou et al., 2021; Rubbab et al., 2022). For instance, Tian et al. (2021) found that individual territorial sense is positively related to information hiding because it involves defensive coping strategies to protect and retain personal information when feeling loss or threat stress. We, therefore, hypothesize as the follow:

*Hypothesis 3: A positive relationship exists between territorial consciousness and subordinate knowledge hiding.*

### The chain-mediating role of job insecurity and territorial consciousness

Small and medium enterprise superiors’ characteristics are reflected in subordinate response behaviors (Steenkamp and Kashyap, 2010). Differential category of SME leadership can have dominating influences on firm functions and shape subordinates’ behavioral choices and decisions (Stamper and Masterson, 2002; Caron et al., 2019). The literature has argued that SME leadership’s effects on subordinates’ response behaviors are transmitted by their perception and awareness (Huang et al., 2010; Mittal and Dhar, 2015; Rehman et al., 2019). Job insecurity and territorial consciousness can act as a chain perception-awareness combination within SMEs. Insecurity from routine work does not mean the passive acceptance of status quos, but engenders strong territorial consciousness by guarding against others who may trespass on a private field (Serenko and Bontis, 2016; Chen et al., 2020). Thus, job insecurity and territorial consciousness bridge SME leadership and subordinate response behaviors; these two constructs cause self-protection on knowledge (Demirkasimoğlu, 2016). For the insiders classified by SME differential leadership, the assigned core tasks trigger uncertainty, tension and pressure from their workplace roles. In addition, more initial resources (vs. outsiders) can also stimulate job crisis awareness and territorialism. In consequence, SME insiders preserve the owned knowledge stocks by rejecting external sharing requests and practicing knowledge hiding behaviors (Anand and Jain, 2014; Tian et al., 2021). For the outsiders defined by SME differential leaders, the perceived risks, threats, powerlessness and worry to maintain desired job continuity can inevitably increase territorialism toward limited knowledge stocks (Feather and Rauter, 2004; Chang and Chuang, 2011; Wang et al., 2015; He et al., 2022).

Based on Hypothesis 1, Hypothesis 2, Hypothesis 3 and the aforementioned arguments, we argue that the effect of SME differential leadership on knowledge hiding is mediated by job insecurity and territorial consciousness coherently. SME differential leaders develop a differentiated superior-subordinate relationship by distinguishing insiders and outsiders, increase the perceived insecurity of both groups in work practices and the sense of territorialism to defend self resources, which in turn promote the unwillingness of subordinates to share information that advance knowledge hiding. Following these discussions, our hypotheses are as follows:

*Hypothesis 4: Job insecurity is positively related to territorial consciousness, and they play a chain mediating role in the relationship between SME differential leadership and subordinate knowledge hiding.*

*Hypothesis 4a: SME differential leadership is positively related to subordinate knowledge hiding.*

*Hypothesis 4b: Job insecurity mediates the positive relationship between SME differential leadership and subordinate knowledge hiding.*

*Hypothesis 4c: Territorial consciousness mediates the positive relationship between SME differential leadership and subordinate knowledge hiding.*

### The moderating role of leadership performance expectation

Social cognitive theory holds a hypothesis of “social-self regulator,” which indicates that individuals try hard to interpret the connotations of environmental cues based on the positioning of social-self, then making behavior plans judiciously (Scott et al., 2022). Leadership performance expectation is a kind of “desired outcomes set by superiors on the basis of given identities within social groups” (Whiteley et al., 2012), which can be understood that employees should maintain a high level of task-oriented attention, increase their concentration and pressure in achieving performance objectives (Mitchell et al., 2018). SME differential leadership is considered in this study as an environmental cue that aggravates the depletion of staff psychological resources due to differential treatment among subordinates (Wang et al., 2018). At the same time, leadership performance expectation creates a challenging work environment with high self-actualized need which can exacerbate the avoidance preference for risk stimuli in the face of SME differential leadership. The manipulative function of employees’ performance maximize objective is inevitable in the framework of social cognitive theory, which lead to subsequent outcome expectations and motivational attributions. In SMEs, the organizational identity of the clue observer allows employees to interpret the hidden information behind differentiated superior management (Wu et al., 2021). Ulteriorly, for employees under higher leadership performance expectation, the initiations of psychological and behavioral reactions are made amidst the social-self stress caused by ongoing concerns about the performance outlook. Then the differentiated human-capital management is more likely to cause a series of negative results. Introducing a moderator is clearly warranted. In this study, we regard leadership performance expectation as social-self regulator, assuming that leadership performance expectation plays a moderating role between SME differential leadership and its outcome variables (e.g., a feeling of unsafety and pessimism about tasks).

The targets of leadership performance expectation are enterprise subordinates who are valued by leaders or accept strict management (Dai et al., 2018). When SME differential leaders set high performance expectation among insiders and outsiders, they may exert high job requirements and thus make both groups feel pressure to pursue excellence (Syrek and Antoni, 2014). As internal and external competition becomes fiercer, SME insiders feel increasingly stressful and nervous to seize success opportunities in core business. Employees regarded as the members of insiders are more likely to engage in performance-enhancing practices and to consider riskier career-development strategies, which often makes them feel depression, anxious and insecurity. On the other hand, because of the embarrassment of resource constraints, SME outsiders suffer both a reduction in physical resources and an increase in negative cognitive ones, and tend to resist interpersonal knowledge interaction. Leadership performance expectation can also lead to these outsiders’ emotional exhaustion and thus to enhancing the sense of resource exhaustion (Li et al., 2019). When SME leaders signal their differential treatment in daily management and resource allocation, individuals who gain sustained high performance expectation perceive more job insecurity. Therefore, we consider a typical superior job anticipation—leadership performance expectation—as a moderating variable in the relationship between SME differential leadership and job insecurity.

According to conservation of resource theory, SME employees with different mentalities reflected by superior job anticipations (i.e., leadership performance expectation) have distinct reaction choices and decisions toward external stimuli (Desrichard and Köpetz, 2005). The nature of leadership performance expectation pilots the crisis awareness of personal knowledge management, decrease the willingness to share knowledge, urge individuals to pay special attention on protecting proprietary and expert information resources, and strengthen the impact of SME differential leadership on knowledge hiding (Qi and Ramayah, 2022). Therefore, this study speculates that leadership performance expectation may also be a prominent moderating factor in the indirect association between SME differential leadership and subordinate knowledge hiding through job insecurity and territorial consciousness. Based on the arguments above, this research posits that when employees are under a higher level of leadership performance expectation, the positive relationship between SME differential leadership and job insecurity is stronger; the knowledge hiding behavior of employees under higher leadership performance expectation is more affected by SME differential leadership *via* job insecurity and territorial consciousness, whereas that of employees under lower leadership performance expectation is less affected by SME differential leadership *via* job insecurity and territorial consciousness. We therefore propose the following hypotheses:

*Hypothesis 5a: The positive relationship between SME differential leadership and job insecurity is stronger when leadership performance expectation is higher.*

*Hypothesis 5b: Leadership performance expectation moderates the indirect relationship between SME differential leadership and subordinate knowledge hiding *via* job insecurity and territorial consciousness, that is, the higher the performance expectation from the leader, the stronger the correlation between SME differential leadership and subordinate knowledge hiding *via* job insecurity and territorial consciousness, and vice versa.*

## Methods

### Sample and procedure

The sample consists of high-tech and manufacturing SMEs located in eastern, southern and middle China (including Guangzhou, Shanghai, Nanjing, Wuhan, Nanchang, Hangzhou, and Fuzhou). Small and medium-sized enterprises exert few constraints on leaders’ discretionary differential treatment toward subordinates (Bauer et al., 2019), superiors play a more pivotal role in affecting employees’ behavior choices and decisions. Hence, this research initially collected data in SMEs with the support of social relationship network, and then we contacted other CEOs in SMEs through the additional recommendations from the familiar CEOs who provided us with valuable information about other SMEs. A total of 70 SMEs (including 140 working teams and 140 leaders) participated in this study.

To greatly decrease common method biases (CMBs), this survey applied a multi-source and multi-period technology to avoid homologous errors (Podsakoff et al., 2003). In the first period (from December 2020 to May 2021), 840 questionnaires were distributed on site in the target small and medium-sized firms. SME employees were requested to rate their superiors’ differential leadership, and to provide information on the own demographics (gender, age, job category, highest education, and how long they have worked with their current leader). We also asked the relevant SME superiors to assess performance expectation of their employees. After matching the completed surveys, 787 sets of questionnaires is effective, with a response rate of 93.69%.

In the second wave (2 months after the first; i.e., from February 2021 to July 2021), to save survey costs and ensure efficiency, all questionnaires were mailed to the target SMEs. Employees in the first wave were asked to assess their own job insecurity, territorial consciousness, and knowledge hiding. A total of 755 questionnaires (89.88%) replied to this second period. After deleting unqualified ones, 704 sets (83.81%) of matched and applicable questionnaires were adopted for statistical hypothesis testing.

The final sample thus includes 704 SME subordinates, 57.95% of whom are classified as males, 42.05% as females (Mean = 0.421; SD = 0.494). Young and middle-aged subordinates comprise most of the final sample, with 86.93% being between the ages of 21 and 50 and 13.07% being above the age of 51 (Mean = 1.348, SD = 0.964). The 40.20% of participants were educated to bachelor degree level, 43.04% to master degree level, 13.64% to doctoral degree level, and 3.12% to postdoctoral degree (Mean = 0.797, SD = 0.789). The jobs of participants are divided into different category groups as follows: functional management (10.80%), R&D (47.87%), product design (36.36%), and market (4.97%) (Mean = 1.355, SD = 0.738). Moreover, with respect to the duration of working with the present leader, 19.60% of participants have worked with their leader for less than or equal to 2 years, 28.69% for a year range of (2, 4), 26.14% for a year range of (4, 6), 25.57% for more than 6 years (Mean = 1.577, SD = 1.072). The details are presented in Table 1.

**TABLE 1:** Sample characteristics (size: 704).

Characteristic	Indicator	Frequency	Percentage	Characteristic	Indicator	Frequency	Percentage
Gender	Male	408	57.95%	Highest education	Bachelor degree	283	40.20%
	Female	296	42.05%		Master degree	303	43.04%
Age	[21–30]	155	22.02%		Doctoral degree	96	13.64%
	[31–40]	241	34.23%		Postdoctoral degree	22	3.12%
	[41–50]	216	30.68%	Duration working with leader	(0, 2)	138	19.60%
	51 and above	92	13.07%		(2, 4)	202	28.69%
Job category	Functional management	76	10.80%		(4, 6)	184	26.14%
	R&D	337	47.87%		Above 6 (excluding)	180	25.57%
	Product design	256	36.36%				
	Market	35	4.97%				

### Measures

Notably, before distributing questionnaires, our research assistants detailed the objective of this research project, informed all respondents of the survey procedure, assured the survey confidentiality, and guaranteed that this survey is irrelevant with their performance evaluation at any stage. To reduce the influence of social desirability, this study then draw the participants’ attention to the importance of honest answers for the sake of this academic survey. To motivate them to participate in the research, every participant who completed the whole survey was given a commemorative reward.

We translated survey questions from English to Chinese following Chen and Boore (2010)’s back-translation procedure to ensure the strictness of questionnaire conversions. In order to ensure consistency, all ratings were given using Likert five-point scales (from “1” = “completely disagree” or “never” to “5” = “strongly agree” or “always”) more details are shown in Appendix.

#### Small and medium enterprise differential leadership

We measured differential leadership adopting a 3-dimension and 14-item scale from Wu et al. (2021) to measure differential treatment as perceived by SME employees. The three dimensions (with item examples) are caring and communication (e.g., “Compared with outsiders, my supervisor seeks out insiders’ opinions on important issues and is biased in communication” and “Compared with outsiders, my supervisor is more sensitive to insiders’ needs and displays more concern for them), promotion and rewards (e.g., “Compared with outsiders, my supervisor cares more about insiders’ priorities, opportunities and self-interests” and “Compared with outsiders, my supervisor provides more incentive pay for insiders), and tolerance of mistakes and faults (e.g., “Compared with outsiders, my supervisor encourages insiders when they make mistakes and faults” and “Compared with outsiders, my supervisor justifies insiders’ mistakes and faults”). The Cronbach’s alpha value is 0.942, showing good reliability. SME differential leadership is a group-level variable. A statistical cross-level test shows high inter-rater agreement among SME employees within each group (average Rwg = 0.917), with ICC (1) and ICC (2) values of 0.346 and 0.727, respectively. These responses were therefore aggregated and used as the reflection and measurement of SME differential leadership.

#### Job insecurity

We adopted a 8-item scale from Hellgren et al. (1999) and Lam et al. (2015) to measure job insecurity from two dimensions (i.e., quantitative job insecurity and qualitative job insecurity). Items include “I feel uneasy about the future of my current job in this firm,” “I raise confusion to think of constructive solutions for issues which appear in my working procedure,” “I am not sure I can reach my goals that may be full of challenge,” “I feel a lack of personal space in the organization,” and “I am nervous to keep my personal resources that may be lost at a faster rate in the future.” The Cronbach’s alpha value of this measure is 0.907, indicating a high level of reliability.

#### Territorial consciousness

The territorial consciousness scale was developed by Avey et al. (2009) and has been widely applied by scholars (e.g., Monaghan and Ayoko, 2019; Wang et al., 2019); it consists of four items. Participants were required to rate each statement, “I think about protecting my present intangible resources from being used by others,” “I think that others should not intrude my workspace,” “I think that others should not use my knowledge, information and ideas without my formal consent,” and “I think I have to inform others not to use the know-how, proprietary knowledge, information and ideas that belong to me,” based on how much they support or deny it. The Cronbach’s α for this scale is 0.858, signifying good reliability.

#### Subordinate knowledge hiding

Subordinate knowledge hiding was rated on a 12-item scale developed by Connelly et al. (2012), by enquiring SME subordinates to evaluate the extent of their intentional acts of concealing the asked individual knowledge by the pattern of pretending ignorance, evasion and giving justifications. Sample items include “When others ask me questions relative to my knowledge, I always say that I do not know, even though I do,” “When others ask me questions related to my knowledge, I always pretend that I do not know or understand what they are talking about,” “When others ask me questions relative to my knowledge, I always agree to help them but instead give them knowledge information different from what they really need,” “When others ask me questions relative to my knowledge, I always promise them that I want to help them later but stall as long as possible,” “When others ask me questions related to my knowledge, I always tell them that the requested knowledge is confidential,” and “When others ask me questions related to my knowledge, I always reflect that I am willing to tell them, but others are unwilling to do so.” Cronbach’s alpha is 0.927, showing high reliability.

#### Leadership performance expectation

The leadership performance expectation scale was originally developed by Gruman and Saks (2011), and verified or revised by some scholars (e.g., Vidyarthi et al., 2014; Mascareño et al., 2020) to align with enterprises in different regions. It consists of three items and participants were asked to rate all statements, “I show this employee that I have high expectations for high-level outcomes from a certain task,” “I show this employee that he or she should finish his or her work efficiently,” “I show this employee that he or she should configure resources most effectively,” based on the extent of the listed expectations implemented by SME leaders. The Cronbach’s α for this scale is 0.864, revealing a high level of reliability.

#### Control variables

This research controlled the impacts of relevant factors, for decreasing possible alternative interpretation for the hypothetical model. We selected and controlled for SME subordinates’ basic demographic characteristics, which have been widely as controls in related literatures (Li et al., 2019). Prior literature has suggested that gender, age, job category, highest education and tenure may affect subordinates’ knowledge hiding (Abdullah et al., 2019; Banagou et al., 2021; Donate et al., 2022; Jasimuddin and Saci, 2022). The researchers have claimed that the aforementioned controls interfere with the interpretive force of the proposed theoretical model. As the demographic information consists of categorical variables, this study measures it after encoding different categories. The control dummy variables include SME subordinates’ gender (“0” = “male,” “1” = “female”; coded with a binary criterion), age (“0” = “[21–30], the reference group,” “1” = “[31–40],” “2” = “[41–50],” “3” = “51 and above”), job category (“0” = “functional management, the reference group,” “1” = “R&D,” “2” = “product design,” “3” = “market”), highest education (“0” = “bachelor degree, the reference group,” “1” = “master degree,” “2” = “doctoral degree,” “3” = “postdoctoral degree”) and the tenure during which they work with their present leader [in years; “0” = “(0, 2), the reference group,” “1” = “(2, 4),” “2” = “(4, 6),” “3” = “above 6 (excluding)”].

## Results

### Reliability and validity of data structure

As demonstrated above, each value of Cronbach’s alpha exceeds the minimum acceptable threshold of 0.700, verifying high reliability of these established scales. The measurement scales have been statistically verified in the research fields, providing support for content validity. To further examine convergent validity of the construct, this study adopted Dutot et al. (2014)’s criteria. The AVE values belonging to SME differential leadership, job insecurity, territorial consciousness, subordinate knowledge hiding, and leadership performance expectation are 0.536, 0.552, 0.603, 0.517, and 0.679, respectively, and all above the threshold of 0.500.

Prior to examining predefined hypotheses, this research performed confirmatory factor analyses (CFAs) on the five self-reported or other-reported scales. We first tested the goodness-of-fit of a 5-factor model that includes SME differential leadership, job insecurity, territorial consciousness, subordinate knowledge hiding, and leadership performance expectation. As anticipated, the proposed 5-factor model demonstrates acceptable fit [χ^2^(769) = 1,414.310, *p* < 0.001; CFI = 0.937; TLI = 0.933; RMSEA = 0.035] with significant standardized loadings ranging from 0.69 to 0.88. The discriminant validity of this five constructs was then demonstrated by contrasting against two alternative constructs: a four-factor model and a single-factor model. The four-factor construct was obtained by loading those items measuring job insecurity and territorial consciousness into a latent factor, since among the five constructs, these two have the highest-level relevance. The single-factor model was gained by loading all items of the five proposed constructs into a latent factor. The CFA test results confirm that the four- and the single-factor models yield poorer goodness-of-fit to the sample data: χ^2^(773) = 2,229.375, *p* < 0.001; CFI = 0.858; TLI = 0.849; RMSEA = 0.052 and χ^2^(779) = 4,698.149, *p* < 0.001; CFI = 0.618; TLI = 0.598; RMSEA = 0.085, respectively. All ratios derived from Δχ^2^ (i.e., changes in χ^2^) divided by Δdf (i.e., changes in df) are significant at *p* < 0.001. Therefore, the fitting effect is ideal; the model construct that makes various items load onto the appropriate factors produces a better fit than any other constructs in which the elements are combined; the five-factor model produces good discriminant validity for our data.

Additionally, the variance contribution rate of the first principal component (36.229%)—an indicator of CMBs—is less than half of the cumulative contribute rate—68.472%, as recommended by Harman’s one factor test, so serious CMB is not present among criterion variables. After adding a method factor [χ^2^(768) = 1,347.840, *p* < 0.001; CFI = 0.943; TLI = 0.940; RMSEA = 0.033], according to the judgment criteria proposed by Min et al. (2016), the fit indexes of the new model is not greatly improved (ΔCFI = 0.006; ΔTLI = 0.007; ΔRMSEA = −0.002). Hence, it can be inferred that common method variance is not a serious issue in the present study.

### Descriptive statistics and correlation analysis

Table 2 reveals the descriptive statistics (i.e., means and standard deviations) and bivariate correlations of all focal variables. The pairwise correlations between the key variables statistically significant and follow the expected relevance direction. SME differential leadership is positively related to job insecurity (*r* = 0.307, *p* < 0.01), territorial consciousness (*r* = 0.266, *p* < 0.01), and subordinate knowledge hiding (*r* = 0.295, *p* < 0.01). Job insecurity is positively and significantly correlated with territorial consciousness (*r* = 0.574, *p* < 0.001), subordinate knowledge hiding (*r* = 0.446, *p* < 0.001) and leadership performance expectation (*r* = 0.209, *p* < 0.01). Territorial consciousness is positively correlated with subordinate knowledge hiding (*r* = 0.505, *p* < 0.001). In addition, subordinate knowledge hiding is positively associated with leadership performance expectation (*r* = 0.309, *p* < 0.01). The correlation results provide preliminary support for the hypotheses.

**TABLE 2:** Descriptive statistics and correlations among focal variables.

Variable	M ± SD	1	2	3	4	5	6	7	8	9
1. Gender	0.421 ± 0.494	−								
2. Age	1.348 ± 0.964	–0.031	−							
3. Job category	1.355 ± 0.738	0.036	0.028	−						
4. Highest education	0.797 ± 0.789	–0.049	–0.075	–0.064	−					
5. Subordinates’ working time with their leader (SWT)	1.577 ± 1.072	0.060	0.081	0.040	–0.097	−				
6. SME differential leadership (SDL)	2.848 ± 0.825	0.015	0.022	–0.011	0.034	−0.138*	−			
7. Job insecurity (JI)	3.166 ± 0.747	0.202**	−0.131*	0.029	0.085	−0.174*	0.307**	−		
8. Territorial consciousness (TC)	3.274 ± 0.836	0.223**	–0.104	0.034	0.111	−0.123*	0.266**	0.574***	−	
9. Subordinate knowledge hiding (SKH)	3.104 ± 0.795	0.168*	−0.172*	0.046	0.156*	–0.102	0.295**	0.446***	0.505***	−
10. Leadership performance expectation (LPE)	3.017 ± 0.929	–0.083	0.193*	0.117*	0.274**	0.182*	0.141*	0.209**	0.352**	0.309**

N = 704; (1) Gender: 0 = male, 1 = female, (2) Age: 0 = 21–30-year-old, 1 = 31–40-year-old, 2 = 41–50-year-old, 3 = 51-year-old and above, (3) Job category: 0 = functional management, 1 = R&D, 2 = product design, and 3 = market, (4) Highest education: 0 = bachelor degree, 1 = master degree, 2 = doctoral degree, 3 = postdoctoral degree, (5) Working time (Time working with their current leader): 0 = (0, 2) years, 1 = (2, 4) years, 2 = (4, 6) years, and 3 = more than 6 years (excluding); ***p < 0.001, **p < 0.01, *p < 0.05.

### Mediating effects of job insecurity and territorial consciousness

We tested our hypotheses on mediating effects using HLM meso-mediational frameworks developed by Mathieu and Taylor (2007) with a cross-level method. To avoid multicollinearity in the mediational effect analysis, the individual-level mediators (job insecurity and territorial consciousness), the moderating variable (leadership performance expectation) were mean-centered in the group level (Zhang et al., 2009). To determine the particular mechanisms by which job insecurity and territorial consciousness mediate the relationship between SME differential leadership and subordinate knowledge hiding, we used the HLM intercept-term equations after adding control variables after centralization. The multicollinearity testing indicates that the values of all of variance inflation factors are <10, indicating that the multicollinearity problem does not exist in this study. The ICC(1) values of the null models on job insecurity (0.189), territorial consciousness (0.215), and subordinate knowledge hiding (0.150) are either greater than or close to the critical value of 0.100, indicating that the data structures are suitable for cross-level analyses.

The results of hypothesis-testing are summarized in Table 3. SME differential leadership is positively related to job insecurity (Model 1: γ05 = 0.271, SE = 0.080, *p* < 0.01) and subordinate knowledge hiding (Model 4: γ05 = 0.257, SE = 0.074, *p* < 0.01), supporting Hypothesis 1 and Hypothesis 4a. Job insecurity has a positive and significant effect on territorial consciousness (Model 3: γ60 = 0.368, SE = 0.110, *p* < 0.01), supporting Hypothesis 2. The results also reveal that the direct influence of territorial consciousness on subordinate knowledge hiding is significant and positive (Model 6: γ70 = 0.343, SE = 0.096, *p* < 0.01), supporting Hypothesis 3.

**TABLE 3:** HLM analysis result.

Fixed-effect	JI		TC	SKH			
			
	Model 1	Model 2	Model 3	Model 4	Model 5	Model 6	Model 7
**Coefficient (SE)**							
Intercept	3.221*** (0.061)	3.207*** (0.067)	3.280*** (0.070)	3.244*** (0.053)	3.252** (0.046)	3.249** (0.049)	3.236*** (0.057)
**Level-1 control variable (individual level)**							
Gender (γ10)	0.103 (0.119)	0.097 (0.102)	0.134^+^ (0.085)	0.081 (0.104)	0.105 (0.121)	0.098 (0.115)	0.088 (0.118)
Age (γ20)	−0.064 (0.084)	−0.072 (0.097)	−0.079 (0.084)	−0.057 (0.075)	−0.078 (0.095)	−0.069 (0.090)	−0.062 (0.083)
Job category (γ30)	0.032 (0.067)	0.046 (0.074)	0.028 (0.053)	0.070 (0.094)	0.090 (0.133)	0.086 (0.107)	0.077 (0.100)
Highest education (γ40)	0.055 (0.082)	0.050 (0.077)	0.064 (0.089)	0.038 (0.057)	0.051 (0.080)	0.049 (0.072)	0.044 (0.061)
SMT (γ50)	−0.087 (0.105)	−0.099 (0.125)	−0.055 (0.076)	−0.051 (0.068)	−0.073 (0.092)	−0.067 (0.085)	−0.059 (0.071)
**Level-1 predictor (individual level)**							
JI (γ60)			0.368** (0.110)		0.292** (0.091)		
TC (γ70)						0.343** (0.107)	0.307** (0.090)
LPE (γ80)		0.394** (0.118)					
**Level-2 control variable (group level)**							
GMJI (γ01)			0.499*** (0.083)		0.301** (0.088)		
GMTC (γ02)						0.421*** (0.064)	0.336** (0.099)
GMLPE (γ03)		0.531*** (0.085)					
SDL × GMLPE (γ04)		0.230** (0.071)					
**Level-2 predictor (group level)**							
SDL (γ05)	0.271** (0.080)	0.248** (0.078)		0.257** (0.074)	**0.056 (0.095)**		**0.069 (0.076)**
**Cross-level interaction effect**							
SDL × LPE (γ81)		0.186* (0.085)					
**Random effect**							
Inter-group variation (τ00)	0.136*** (110.446)	0.061** (88.195)	0.177*** (118.505)	0.118*** (103.694)	0.051** (80.216)	0.104** (97.988)	0.098** (96.173)
Slope variance of JI (τ66)			0.042 (76.563)		0.014 (61.048)		
Slope variance of TC (τ77)						0.035 (73.126)	0.029 (68.726)
Slope variance of LPE (τ88)		0.037 (74.592)					
Intra-group variation (σ^2^)	0.763	0.745	0.772	0.742	0.478	0.651	0.640
−2 Log likelihood (−2LL)	751.459	732.607	780.228	737.885	625.229	720.015	717.894

N = 704; (1) GMJI: group mean of job insecurity, (2) GMTC: group mean of territorial consciousness, (3) GMLPE: group mean of leadership performance expectation; ***p < 0.001, **p < 0.01, *p < 0.05, ^+^p < 0.10.

Since the three transmittal effects are statistically significant, this research examined whether the mediating effects were significantly present in terms of job insecurity and territorial consciousness. The results show that job insecurity is positively related to subordinate knowledge hiding (Model 5: γ60 = 0.292, SE = 0.091, *p* < 0.01), and the correlation between SME differential leadership and subordinate knowledge hiding is much weaker than that in Model 4 (Model 4: γ05 = 0.257, SE = 0.074, *p* < 0.01; Model 5: γ05 = 0.056, SE = 0.095, n.s.). The mediating effect of job insecurity exists, supporting H4b. It can be found that the correlation between territorial consciousness and subordinate knowledge hiding (Model 7: γ70 = 0.307, SE = 0.090, *p* < 0.01) is positive and significant. Similarly, there was a significant difference between the SME differential leadership—subordinate knowledge hiding relationship in Model 7 than that in Model 4 (Model 4: γ05 = 0.257, SE = 0.074, *p* < 0.01; Model 7: γ05 = 0.069, SE = 0.076, n.s.), which further supported H4c, that is, the influence of SME differential leadership on subordinate knowledge hiding is mediated by territorial consciousness.

We created four serial competition models (i.e., incomplete dual mediating model, complete dual mediating model 1, complete dual mediating model 2, single chain-mediating model) in Table 4, which are all inferior to the full model in goodness of fit: χ^2^(659) = 1,217.143, *p* < 0.001; CFI = 0.947; TLI = 0.944; RMSEA = 0.035; SRMR = 0.020, and the values of Δχ^2^/Δdf test are significant at the level of 0.001, indicating that the full chain-mediating model is the most suitable construct obtained for latent variables.

**TABLE 4:** Model fitting result.

Model	Graphic description	χ ^2^	df	χ ^2^/df	Δχ ^2^/Δ df	CFI	TLI	RMSEA	SRMR
Full chain-mediating model	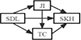	1217.143	**659**	**1.847**	**—**	**0.947**	**0.944**	**0.035**	**0.020**
Incomplete dual mediating model	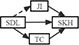	1530.759	660	2.319	313.616***	0.917	0.912	0.043	0.032
Complete dual mediating model 1	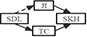	1575.310	661	2.383	179.084***	0.913	0.908	0.044	0.039
Complete dual mediating model 2	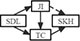	1324.085	660	2.006	106.942***	0.937	0.933	0.038	0.026
Single chain-mediating model	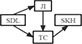	1304.726	661	1.974	43.792***	0.939	0.935	0.037	0.035

N = 704; Dashed lines mean that this path is not significant; ***p < 0.001, two-tailed test.

Then, the mediating effect test results from 5,000 Bootstrap samples are shown in Table 5: SME differential leadership has a significant indirect impact on subordinate knowledge hiding through job insecurity (effect = 0.047, SE = 0.018, 95% CI: [0.011, 0.084]), this mediation effect (SME differential leadership → job insecurity → subordinate knowledge hiding) accounted for 17.21% of the total effect, supporting 4b; territorial consciousness mediates the relationship between SME differential leadership and subordinate knowledge hiding (effect = 0.055, SE = 0.020, 95% CI: [0.018, 0.092]), this mediation effect (SME differential leadership → territorial consciousness → subordinate knowledge hiding) accounted for 20.15% of the total effect, supporting 4c; finally, the indirect effect of SME differential leadership on subordinate knowledge hiding through job insecurity and territorial consciousness (i.e., a chain mediating effect) is also found (effect = 0.020, SE = 0.009, 95% CI: [0.007, 0.034]), this chain mediation effect (SME differential leadership → job insecurity → territorial consciousness → subordinate knowledge hiding) accounted for 7.33% of the total effect. Therefore, we believe that job insecurity is positively related to territorial consciousness, and they play a chain mediating role between SME differential leadership and subordinate knowledge hiding, which supports H4.

**TABLE 5:** Chain-mediating effect analysis.

Effect and path	Estimated value	SE	95% LLCI	95% ULCI	Proportion
Direct effect: SDL → SKH	0.151***	0.035	0.039	0.265	55.31%
Total mediating effect	0.122**	0.038	0.026	0.219	44.69%
**Decomposition of total mediating effect**					
Independent mediating path 1 (IMP 1): SDL → JI → SKH	0.047*	**0.018**	**0.011**	**0.084**	**17.21%**
IMP 2: SDL → TC → SKH	0.055*	**0.020**	**0.018**	**0.092**	**20.15%**
IMP 3: SDL → JI → TC → SKH	0.020*	**0.009**	**0.007**	**0.034**	**7.33%**
Total effect: SDL → SKH	0.273***	0.055	0.162	0.386	100.00%

N = 704; ***p < 0.001, **p < 0.01, *p < 0.05; Bootstrap based on repeating sampling 5000 times.

**FIGURE 1:**
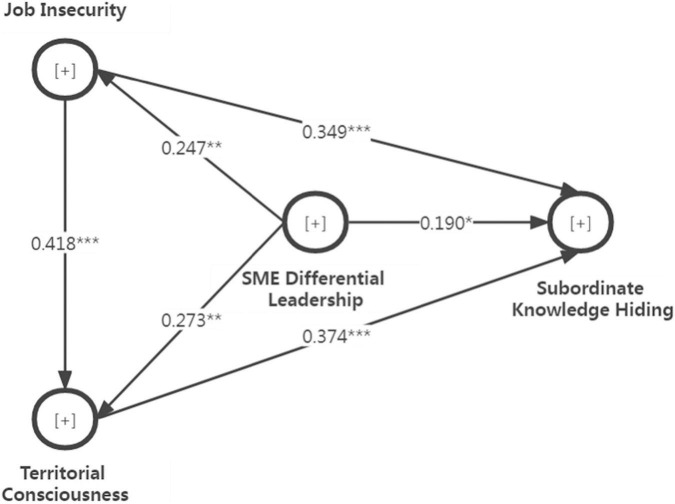
Hypothetical full model diagram: job insecurity and territorial consciousness play a chain mediating role on the relationship between SME differential leadership and subordinate knowledge hiding. ***p < 0.001, **p < 0.01, *p < 0.05.

In addition, we tested the path coefficients of the full chain-mediating model, which consists of three indirect impacts: (a) SME differential leadership increases subordinate knowledge hiding *via* job insecurity, (b) SME differential leadership increases subordinate knowledge hiding *via* territorial consciousness, and (c) SME differential leadership increases subordinate knowledge hiding *via* job insecurity and territorial consciousness in Figure 1 where the chain mediating path is verified. The direct impact of SME differential leadership on job insecurity is significant (*B* = 0.247, *p* < 0.01). The results show a positive effect of job insecurity on territorial consciousness (*B* = 0.418, *p* < 0.001) and a positive effect of job insecurity on subordinate knowledge hiding (*B* = 0.349, *p* < 0.001). There is a positive and significant relation between SME differential leadership and territorial consciousness (*B* = 0.273, *p* < 0.01). Both SME differential leadership and territorial consciousness significantly predict subordinate knowledge hiding (*B* = 0.190, *p* < 0.05, for SME differential leadership; *B* = 0.374, *p* < 0.001, for territorial consciousness). According to the impact of SME differential leadership on subordinate knowledge hiding when job insecurity and territorial consciousness play a parallel mediating role, it can be testified that the indirect correlation between SME differential leadership and subordinate knowledge hiding (*B* = 0.150 = 0.247 × 0.349 + 0.273 × 0.374 – 0.247 × 0.418 × 0.374) and the total impact between that (*B* = 0.340 = 0.190 + 0.150) are both positive, that is, the influence of SME differential leadership on subordinate knowledge hiding is mediated by the chain effect from job insecurity and territorial consciousness.

### Moderating effects of leadership performance expectation

**FIGURE 2:**
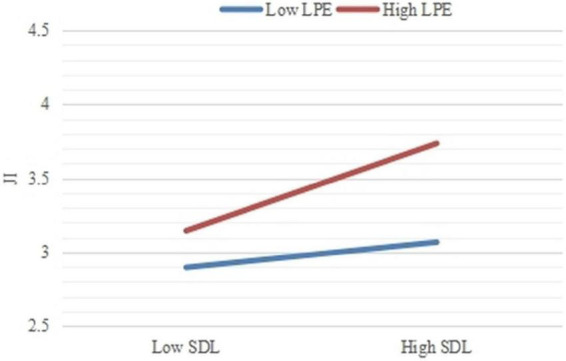
The moderating role of leadership performance expectation on the relationship between SME differential leadership and job insecurity.

**FIGURE 3:**
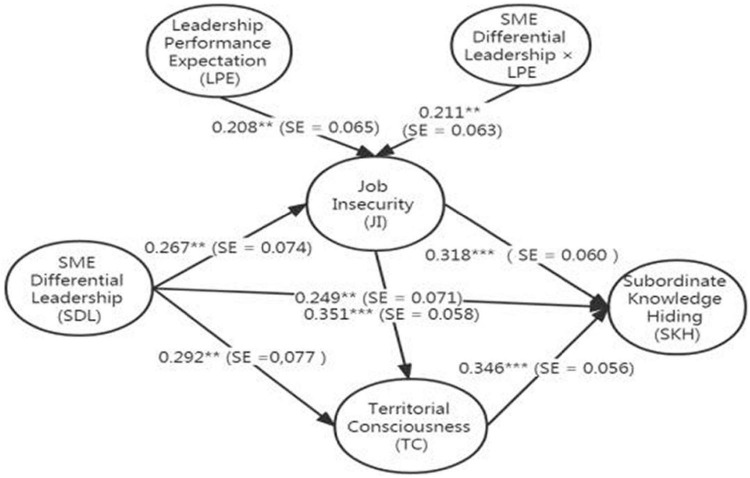
Correlation coefficients of the full model. ***p < 0.001, **p < 0.01.

Hypothesis 5a predicts that leadership performance expectation positively moderates the direct relationship between SME differential leadership and job insecurity, and Hypothesis 5b predicts that leadership performance expectation positively moderates the indirect relationship between SME differential leadership and subordinate knowledge hiding through job insecurity and territorial consciousness. Table 3 shows that the interaction term of SME differential leadership with performance expectation from leaders is significantly and positively related to job insecurity (Model 2: γ81 = 0.186, SE = 0.085, *p* < 0.05). This finding supports H5a. To reflect the nature of this moderating effect, this research plotted the interaction effects by calculating slopes one standard deviation above and below the mean of leadership performance expectation. Figure 2 shows that for SME subordinates with a higher level of leadership performance expectation, the positive relationship between SME differential leadership and job insecurity is stronger (simple slope = 0.358, *p* < 0.001) compared to SME subordinates with a lower level of leadership performance expectation (simple slope = 0.104, *p* = 0.112, n.s.). The technique of orthogonal product index (i.e., standardized path coefficients corrected according to the STDYX coefficients provided by Mplus 7.4) is adopted for constructing interaction terms when analyzing the moderation effects of leadership performance expectation. The orthogonalizing technique minimizes estimation bias in the respect of point accuracy, yielding good prediction accuracy. Figure 3 reveals that the interaction term of SME differential leadership and leadership performance expectation on job insecurity is positive and significant (β = 0.211, SE = 0.063, *p* < 0.01).

Finally, the moderating role of leadership performance expectation in the cross-level chain-mediating effect is tested. To more directly assess the moderating effect of leadership performance expectation, a residual Bootstrap method with non-parametric percentile for error correction is used. The analyses on 5,000 Bootstrap samples in Table 6 indicate that the indirect chain-mediating effect of SME differential leadership on subordinate knowledge hiding is stronger for higher leadership performance expectation than for lower leadership performance expectation (SME differential leadership—higher leadership performance expectation: β = 0.049, SE = 0.026, 95% CI [0.030, 0.071], lower leadership performance expectation: β = 0.022, SE = 0.011, 95% CI [0.004, 0.039]). There is a significant difference in the chain mediation correlation between SME differential leadership and subordinate knowledge hiding between high performance expectation group and low performance expectation group (β = 0.027, SE = 0.014, 95% CI [0.006, 0.050]), which supports H5b.

**TABLE 6:** Cross-level analysis on moderated chain-mediating effect.

	Chain-mediating path: SDL → JI → TC → SKH					
	
Moderator	Phase			Effect		
		
	Phase I: Psj SDL → JI (SE)	Phase II: Pjt JI → TC (SE)	Phase III: Pts TC → SKH (SE)	Direct effect: Pss SDL → SKH (SE)	Indirect chain-mediating effect: Psj * Pjt * Pts SDL → JI → TC → SKH (SE)	Total effect: Pss + Psj * Pjt * Pts (SE)
High LPE (Mean + 1 SD = 3.946)	0.329*** (0.247, 0.410)	0.380*** (0.349, 0.412)	0.391*** (0.327, 0.454)	0.283*** (0.234, 0.333)	0.049* **(0.030, 0.071)**	0.332*** (0.259, 0.406)
Low LPE (Mean – 1 SD = 2.088)	0.222** (0.138, 0.305)	0.334*** (0.305, 0.364)	0.302*** (0.240, 0.365)	0.218** (0.167, 0.270)	0.022* **(0.004, 0.039)**	0.240** (0.165, 0.316)
Difference	0.107** (0.027, 0.186)	0.046* (0.013, 0.080)	0.089** (0.039, 0.148)	0.065** (0.012, 0.117)	0.027* **(0.006, 0.050)**	0.092** (0.019, 0.164)

N = 704; ***p < 0.001, **p < 0.01, *p < 0.05; Bootstrap based on repeating sampling 5000 times.

## Discussion

The impact of differential leadership on employees’ reactions has attracted the attention of a number of previous scholars (Stamper and Masterson, 2002; Knapp et al., 2014; Dai and Chen, 2015; Wang et al., 2018; Pervaiz et al., 2021; Wu et al., 2021; Metz et al., 2022), who expressed different study opinions. Although a few of the existing researches have studied the correlations among differential leadership and non-prosocial behaviors (Wang et al., 2018), making certain theoretical contributions from the relationship element framework, relatively few papers exist on detailed analyses of differential leadership on individual knowledge hiding through trickle-down effects. Therefore, this research empirically examines whether and how SME differential leadership leads to subordinate knowledge hiding.

Furthermore, the extant literature has represented knowledge hiding as a relationship-management construct with respect to personal intellectual capital (Demirkasimoğlu, 2016; Qi and Ramayah, 2022). Namra et al. (2021) summarized previous literature and proposed that individual personalities, interpersonal interactions, reciprocity norms and organizational climate can lead to employees’ knowledge hiding. Through a systematic literature review and analysis, this research finds that, although some studies explore antecedents of knowledge hiding from individual-level element perspectives, such as personality traits (Anand and Jain, 2014; Demirkasimoğlu, 2016), and psychological characteristics (Ain et al., 2021; Tian et al., 2021), these existing studies scarcely explores knowledge hiding from the view of higher-up management features. The most recent literature has continuously explore knowledge hiding in relation to contemporary leadership phenomena, occurring frequently in the current era of “post-COVID-19” (Batistic and Poell, 2022), such as insufficient resources available for allocation (Corwin et al., 2022) or clubbing together in workplace (Jasimuddin and Saci, 2022). Although existing theoretical books, reviews, and papers have widely examined and highlighted the impacts of various types of SME leadership on a series of subordinate behaviors, most, to our knowledge, have largely ignored the differential category of SME leaders and have seldom observed or analyzed the influence mechanisms of this leadership category, especially specific influences on individual knowledge-based actions. We rigorously introduce the construct of SME differential leadership into the academic field of individual knowledge hiding, theoretically developing the formation explanation of knowledge hiding in the context of a typical SME leadership style.

However, the study of the differential leadership—knowledge hiding relevance from the perspective of double central processes of perception and consciousness has not been discussed. We studied internal processes that SME differential leadership has impacts on knowledge hiding on the basis of job insecurity and territorial consciousness, further elaborating intrinsic mechanisms that SME differential leadership increases knowledge hiding. The existing research explores the internal mediating mechanisms of knowledge hiding, from the perspective of Islamic work ethics (Khalid et al., 2018), relational social capital (Abdullah et al., 2019), and unlearning (Cegarra-Navarro et al., 2021). More efforts are needed to provide comprehensive research on generation mechanisms toward knowledge hiding behavior. SME differential leadership that distribute work and emotional resources unequally is important for driving employees to perceive job insecurity. It can also produce the possible positive effect on territorial consciousness among SME individuals, make them induce stronger ownership, simplify individuals’ social interactions to strengthen the object-belongingness. In fact, although SME differential leadership guides employees to actively select avoidance-coping strategies and better retain individual resources, due to the actual state of feeling loss or threat of loss from workplace stressors in the process of subordinate knowledge hiding, subordinate knowledge hiding may be more stimulated by job insecurity and territorial consciousness. In our paper, the impact mechanisms of “SME differential leadership—job insecurity—territorial consciousness—knowledge hiding” indicates the chain formation of subordinate knowledge hiding and can better help others understand inner process from SME differential leadership on knowledge hiding.

Existing literature focuses on the perspective of organizational justice (Tang et al., 2018), within-team mean degree and the within-team variance degree (Wang et al., 2018), and proactive personality (Zhang et al., 2022) when exploring the moderation for the relationship between differential leadership and the outcome variables. However, prior studies lack consideration from the perspective of leadership stressor. Although the benefits of leadership performance expectation are many, challenges also exist, including devoting a considerable amount of time and energy achieving high work achievement, which is likely to increase individuals’ obstructive stress. We apply social cognitive theory to the contextual effect of leadership-based stress—leadership performance leadership performance expectation on both job insecurity and knowledge hiding, addressing the previous call that “more theoretical and empirical work is really needed to explore the respective coping strategies toward knowledge hiding” (proposed by Rubbab et al., 2022).

### Theoretical implications

This study expounds on theoretical implications, following the logic of theoretical composition, including central relationship, mediating mechanisms, and moderating mechanisms. First, this research expands the body of emerging SME leadership research and elucidates the central process of the influence mode of SME differential leadership on personal knowledge management. When SME staff realize different treatments from their superiors, they gradually sense that they have more (for insiders) or less (for outsiders) value and significance within this organization, leading to their tendency to keep knowledge as an exclusive tool (Dai and Chen, 2015; Peng and Pierce, 2015; Banagou et al., 2021; Pervaiz et al., 2021). Thus, our research has promoted a broader theoretical perspective of differential leadership by highlighting the potential impact process on knowledge hiding that previous study has ignored. This study complements literature on how SME differential leadership results in individual behavioral decisions and enriches the differential leadership—knowledge hiding interface study. This research applies conservation of resource theory and social cognitive theory to SME situations involving differential leadership in order to expand its application scope.

Second, more specifically, we conduct more in-depth analyses on the differential leadership—knowledge hiding modeling. This research demonstrates that SME differential leadership can shape personal knowledge management not only by forming a perception of workplace insecurity (Dai and Chen, 2015; Stankevičiûtë et al., 2021), but also by arousing spontaneous and proactive self-defense and territorial strategies for retaining knowledge (Banagou et al., 2021; Tian et al., 2021). The findings suggest that the constant change/upgrade bring normal uncertainty of employees in SMEs. By emphasizing common and imperceptible workplace psychological problems, SME differential leadership can promote job insecurity as a “by-product” of daily work and motivate employees to deal with their threat and pressure in their working environment, which translates to enhanced subordinate knowledge hiding. Based on relative deprivation theory, SME employees gain a stronger sense of territorial nature of resources when they have no countermeasure to change leaders’ differential mode of thinking (Rubbab et al., 2022). The possibility of intentional acts of concealing the asked information or knowledge through individual territorial consciousness increases in the face of differential leadership. Resource conservation research states that effects of SME leadership on employee responses can be transmitted by two dynamic mechanisms (job insecurity or territorial consciousness) at psychological level. Therefore, in this study, we introduce job insecurity and territorial consciousness in the intervening mechanisms and verify their mediating roles on how SME differential leadership influences subordinate knowledge hiding. Our study paves the way for future empirical researches to investigate whether differential leadership causes certain individual-level consequences in SMEs.

Third, the results show that job insecurity and territorial consciousness have a chain mediating effect on the correlation between SME differential leadership and subordinate knowledge hiding. This result echoes the theory of constructivism and reveals that sense and awareness can have a chain mediating influence on the relationship between schema shaped by environmental events and behaviors (Chen et al., 2021a). Specifically, SME differential leadership, by increasing subordinates’ ego depletion, guides insiders and outsiders to balance internal pressure and external surroundings. Employees in a highly stressed condition may feel more job insecurity, and the cumulative effect will cause an increasingly strong unwillingness to share privately owned resources, consequently leading to knowledge hiding (Demirkasimoğlu, 2016; Ain et al., 2021; Batistic and Poell, 2022). The interplay between job insecurity and territorial consciousness provides a strong explanation on how SME leaders’ differentiation style influences the occurrence of subordinate knowledge hiding, supporting the core opinion of conservation of resource theory that higher resource depletion or shortage is likely to cause a state of stress and worry, therefore resulting in resource conservation intentions.

Finally, this paper contributes to research on the linkage between SME differential leadership and job insecurity by identifying one typical conditional factor—leadership performance expectation. The interaction between SME differential leadership and leadership performance expectation can result in a stronger explanation of the heterogeneity of subordinates’ job insecurity. Although Oubrich et al. (2021) have demonstrated that leadership styles have a possibility to induce the intention to hide knowledge, they do not investigate boundary conditions that may place limits on how organizational leadership actually influences subordinates’ unwillingness to share their knowledge information. Leadership performance expectation, as a prominent detail that reflects more resources to be acquired and invested during work process, affects their interpretations of, and reactions to stimuli from surrounding environment (Gruman and Saks, 2011). SME employees who are under higher performance expectations from their leaders are more sensitive to differential leadership, feel insecurity in work tasks, and divide the territory for self resources. In this manner, we create a theoretical model of when SME differential leadership facilitates subordinates’ job insecurity and territorial consciousness which then advances knowledge hiding. Therefore, we push this study forward by introducing the level of leadership performance expectation as one representative boundary condition, which significantly moderates the indirect relationship between SME differential leadership and subordinate knowledge hiding through a chain-mediating path from job insecurity to territorial consciousness. The contextual exploration on leadership performance expectation can extend the understanding on how to aggravate the negative effect of SME differential leadership.

### Practical implications

Our conclusions also have significant implications for SME management practitioners. Accelerated technological change and competition make individual willingness to interact effective knowledge with others a must for enterprises’ sustainable competitiveness (Lee et al., 2021; Vătămănescu et al., 2022). Owning unchanging knowledge can not form stable core competitiveness of people, constantly sharing and absorbing knowledge is an important foundation for realizing value additions. However, the engagement in knowledge sharing cannot be achieved only by individual spontaneity. It is a synthetic process that covers external environmental factors and internal individual characteristics together. Therefore, it appears important for SME superiors to promote knowledge connections and interactions among organizational employees. Differential leadership is highly relevant to practices on sharing private knowledge. This research provides evidence that SME differential leadership promotes subordinate knowledge hiding *via* job insecurity and territorial awareness. Thus, to impede knowledge hiding, SME leaders should reduce unbalance-management behaviors (highly valued or dismissive) and pay attention to relationship maintenance with subordinates.

Additionally, the mediating mechanism of job insecurity reaffirms that SME superiors should encourage their employees to overcome occupational insecurity by providing timely psychological counseling and team building activities. By observing and caring employees’ psychological insecurity state, corresponding measures need to be implemented to guide them toward an uplifting and positive mind state; thus, SME leaders should create a relatively fair atmosphere to establish a harmonious work-related platform with stable characteristics. Simultaneously, this finding also reaffirms that apart from providing security support, SME superiors should also pay attention to the ideology of individual interactions across territories. It is imperative to deepen understandings of employees to promote benign interactions. Even on a small or medium size, enterprises that emphasize innovation should inspire employees to not only exchange information, know-how, experiences, opinions and ideas with others, but also to break through narrow workspace to conduct frank and open communication.

Lastly, our study creatively highlights that leadership performance expectation is an important boundary condition in both a direct relation (SME differential leadership → job insecurity) and a indirect one with a chain-mediating effect (SME differential leadership → job insecurity → territorial consciousness → subordinate knowledge hiding). SME executives should set performance targets for employees in consistency with their own expectations rather than establishing unrealistic and blind ones. This is especially necessary in the VUCA era, as SME technology capability becomes more prior and subordinate knowledge sharing becomes an effective way which benefits overall organizational interests. Additionally, SME staff are more likely to feel stressed, tired and burned out to deal with complicated situations when faced with higher leadership performance expectation (Dai et al., 2018). When stressed subordinates are assigned job tasks, we remind SME managers to set performance expectations in a reasonable range to reduce its possible negative impacts.

### Limitations and future research

This research has some limitations, which we realize should be address in future literature. First, the generalization of the relationship among SME differential leadership, job insecurity, territorial consciousness, subordinate knowledge hiding, and leadership performance expectation should be verified in distinct regional backgrounds; we collected research data from 70 Chinese SMEs and demonstrated the effects of SME differential leadership only in the Chinese context. China is considered a link with a traditional culture of Confucianism (Metz et al., 2022), so it provides an appropriate context to confirm the effects of differential leadership. In order to improve generalizability of our findings, a novel extension of this research is to empirically explore whether and how the impacts of SME differential leadership differ in eastern and western cultural values. In addition, the outcomes may be more applicative in technological enterprises, while the universality of these results remains unclear within trade- or service-oriented SMEs. In accordance with the suggestion from Chen et al. (2021b), future researchers can also examine our model with a wider scope of participants to allow greater external validity in more diverse and emerging industries.

Second, we primarily capture a complex mechanism of subordinate knowledge hiding. Although all scales have perfect reliability and validity, our claim of causality does not have enough persuasive power; that is, the measurement of job insecurity, territorial consciousness, knowledge hiding and leadership performance expectation relied on self-report. It may cause social desirability issues because of the possibility of unrealistic positive responses, which go against data objectivity. Future study may consider not only selecting multiple informants but also adopting more objective methodologies in this long-term follow-up surveys. Therefore, more effective, accurate, and reliable results may be obtained.

Third, this paper only focuses on impacting factors of subordinate knowledge hiding from perspectives of leadership styles, sensations and consciousness based on conservation of resource theory and social cognitive theory. Meanwhile, SME differential leadership may also lead to a series of consequences (e.g., intra-team innovation ability at the organizational level, and job involvement at the individual level) that warrant more empirical verification. Future study should either introduce more categories of factors into the model or validate the statistical modeling to a broader level to test other mechanisms by which subordinate knowledge hiding is influenced or Differentiated leadership exerts distinct impacts.

Finally, this research identifies job insecurity and territorial consciousness as mediators, as these two mental constructs represent a typical perceptual consciousness. However, we have not directly considered SME employees’ general cognition traits. Although an adoption of proxy variables is a common research practice, recent systematic reviews on this practice (Clark and Plano Clark, 2019; Calderwood et al., 2020) have pointed out the faultiness of using proxy variables to capture actual psychological and cognitional reality. While questionnaire respondents showing distinct psychological features may react differently to SME differential management which impacts their willingness to conceal knowledge, as Verhulst et al. (2019) found that neuro-theories and neuroscience tools in the leadership field can better explain the appearance of knowledge hiding. Hence, a potentially meaningful extension of our research would be to affirm the complex effect of SME differential leadership on subordinate knowledge hiding by directly using neuro-tools.

## Conclusion

On the basis of the 704 questionnaires of this two-wave survey, our empirical analyses provide a novel result: SME differential leadership can positively predict subordinate knowledge hiding. Through the analysis of the full model, we found that job insecurity and territorial consciousness jointly play a positive chain-mediating role in this influence process. This research also discusses the association between SME differential leadership and job insecurity is positively moderated by leadership performance expectation. We believe that the mediating roles of job insecurity and territorial consciousness between differential leadership in SMEs and subordinate knowledge hiding includes three paths: the independent and chain mediation of job insecurity and territorial consciousness. SME differential leadership causes individuals to perceive negative workplace powerlessness, feel threatened to maintain required status, and stimulate more obstructive stress and job insecurity, so as to provide antecedents for individual territorial consciousness and then promote knowledge hiding of subordinates.

One finding is the moderating effect of leadership performance expectation on the direct correlation between SME differential leadership and job insecurity. Specifically, when a SME employee experiences stress due to relatively high expectations, the feeling of uncertainty is enhanced, leading to increased fear, which will easily result in a threat of losing current work continuity, thus driving a insecure perceptual pattern (Staufenbiel and König, 2010; Smith et al., 2012; Li et al., 2014). Therefore, the correlation between SME differential leadership and job insecurity is strengthened by the increase of leadership performance expectation. Similarly, the result verifies the hypothesis of the present study that leadership performance expectation enhances a psychological state that includes both insecurity and territorialism, and its significant moderating effect on subordinate knowledge hiding needs to be realized *via* the chain mediation of other elements (i.e., job insecurity and territorial consciousness). When a SME employee is exposed to a stressful condition, he or she will be more prone to one or more consciousness that induce adaptive actions. Driven by performance expectations from differential leaders, SME subordinates are more likely to adopt knowledge hiding affected by their job insecurity and territorial consciousness.

## Data availability statement

The original contributions presented in this study are included in the article/supplementary material, further inquiries can be directed to the corresponding author.

## Ethics statement

The protocol for this current research was reviewed and approved by the Institutional Review Board of Department of Evergrande School of Management at Wuhan University of Science and Technology. All participants provided their written informed consent to participate in this study.

## Author contributions

JX contributed to conducting data analysis and editing the final manuscript as submitted. DZ generated the study idea with the theoretical development of the research. YL was responsible for the structure and content. All authors contributed to the article and approved the submitted version.

## Appendix

SME Differential Leadership (Wu et al., 2021; the dimension of caring and communication: SDL1—SDL4; the dimension of promotion and rewards: SDL5—SDL9; the dimension of tolerance of mistakes and faults: SDL10—SDL14).

SDL1: Compared with outsiders, my supervisor seeks out insiders’ opinions on important issues and is biased in communication.
SDL2: Compared with outsiders, my supervisor is more sensitive to insiders’ needs and displays more concern for them.
SDL3: Compared with outsiders, my supervisor spends more time guiding insiders at work.
SDL4: Compared with outsiders, my supervisor assigns insiders to convey important messages.
SDL5: Compared with outsiders, my supervisor cares more about insiders’ priorities, opportunities and self-interests.
SDL6: Compared with outsiders, my supervisor provides more incentive pay for insiders.
SDL7: Compared with outsiders, my supervisor assigns insiders simple jobs which can easily succeed.
SDL8: Compared with outsiders, my supervisor offers or reserve the opportunity of potential promotion opportunities for insiders.
SDL9: Compared with outsiders, my supervisor gives insiders a rapid promotion.
SDL10: Compared with outsiders, my supervisor helps insiders in case of emergency.
SDL11: Compared with outsiders, my supervisor encourages insiders when they make mistakes and faults.
SDL11: Compared with outsiders, my supervisor gives insiders a lighter punishment when they make mistakes and faults.
SDL12: Compared with outsiders, my supervisor justifies insiders’ mistakes and faults.
SDL13: Compared with outsiders, my supervisor is more likely to turn a blind eye to the mistakes made by insiders.
SDL14: Compared with outsiders, my supervisor is less likely to blame and pursue insiders for their mistakes and faults at work.

**Job Insecurity (Hellgren et al., 1999 and Lam et al., 2015; the dimension of qualitative job insecurity: JI1—JI4; the dimension of quantitative job insecurity: JI5—JI9)**

JI1: I feel uneasy about the future of my current job in this firm.
JI2: I raise confusion to think of constructive solutions for issues which appear in my working procedure.
JI3: The lack of security in the company makes me nervous.
JI4: Hard work does not guarantee my present job.
JI5: I am afraid I am not competent for my current job and bear the risk of leaving the post.
JI6: I am not sure I can reach my goals that may be full of challenge.
JI7: I feel a lack of personal space in the organization.
JI8: I am nervous to keep my personal resources that may be lost at a faster rate in the future.

**Territorial Consciousness (Avey et al., 2009)**

TC1: I think about protecting my present intangible resources from being used by others.
TC2: I think that others should not intrude my workspace.
TC3: I think that others should not use my knowledge, information and ideas without my formal consent.
TC4: I think I have to inform others not to use the know-how, proprietary knowledge, information and ideas that belong to me.

**Subordinate Knowledge Hiding (Connelly et al., 2012; the dimension of pretending ignorance: SKH1—SKH4; the dimension of evasion: SKH5—SKH8; the dimension of giving justifications: SKH9—SKH12)**

SKH1: When others ask me questions relative to my knowledge, I always say that I do not know, even though I do.
SKH2: When others ask me questions related to my knowledge, I always pretend that I do not know or understand what they are talking about.
SKH3: When others ask me questions related to my knowledge, I always say that I do not know much about this topic.
SKH4: When others ask me questions relative to my knowledge, I always say that I only know the surface of this topic.
SKH5: When others ask me questions relative to my knowledge, I always agree to help them but instead give them knowledge information different from what they really need.
SKH6: When others ask me questions relative to my knowledge, I always promise them that I want to help them later but stall as long as possible.
SKH7: When others ask me questions related to my knowledge, I always tell them some other information instead of what they really want to know.
SKH8: When others ask me questions related to my knowledge, I always agree their request, but I will not actually respond.
SKH9: When others ask me questions related to my knowledge, I always tell them that the requested knowledge is confidential.
SKH10: When others ask me questions related to my knowledge, I always reflect that I am willing to tell them, but others are unwilling to do so.
SKH11: When others ask me questions related to my knowledge, I always reflect that my leader has ordered me not to impart heterogeneous knowledge.
SKH12: When others ask me questions related to my knowledge, I always tell them that I think the requested knowledge is almost useless to them.

**Leadership Performance Expectation (Gruman and Saks, 2011)**

LPE1: I show this employee that I have high expectations for high-level outcomes from a certain task.
LPE2: I show this employee that he or she should finish his or her work efficiently.
LPE3: I show this employee that he or she should configure resources most effectively.
